# Oxaliplatin combined with infusional 5-fluorouracil and concomitant radiotherapy in inoperable and metastatic rectal cancer: a phase I trial

**DOI:** 10.1038/sj.bjc.6602413

**Published:** 2005-02-08

**Authors:** S Loi, S Y K Ngan, R J Hicks, B Mukesh, P Mitchell, M Michael, J Zalcberg, T Leong, D Lim-Joon, J Mackay, D Rischin

**Affiliations:** 1 Division of Haematology and Medical Oncology, Peter MacCallum Cancer Centre, Melbourne, Victoria, Australia; 2 Division of Radiation Oncology, Peter MacCallum Cancer Centre, Melbourne, Victoria, Australia; 3 Centre for Molecular Imaging, Peter MacCallum Cancer Centre, Melbourne, Victoria, Australia; 4 Centre for Biostatistics and Clinical Trials, Peter MacCallum Cancer Centre, Melbourne, Victoria, Australia; 5 Cancer Services, Austin and Repatriation Hospital, Heidelberg, Victoria, Australia; 6 Division of Surgical Oncology, Peter MacCallum Cancer Centre, Melbourne, Victoria, Australia

**Keywords:** chemoradiotherapy, oxaliplatin, positron emission tomography, rectal cancer, hypoxia

## Abstract

The aim of this study was to define the recommended dose of oxaliplatin when combined with infusional 5-fluorouracil (5-FU) and concurrent pelvic radiotherapy. Eligible patients had inoperable rectal cancer, or symptomatic primary rectal cancer with metastasis. Oxaliplatin was given on day 1 of weeks 1, 3 and 5 of radiotherapy. Dose level 1 was oxaliplatin 70 mg m^−2^ with 5-FU 200 mg m^−2^ day^−1^ continuous infusion 96 h week^−1^. On dose level 2, the oxaliplatin dose was increased to 85 mg m^−2^. On dose level 3, the duration of the 5-FU was increased to 168 h per week. Pelvic radiotherapy was 45 Gray (Gy) in 25 fractions over 5 weeks with a boost of 5.4 Gy. Fluorine-18 fluoro deoxyglucose and Fluorine-18 fluoro misonidazole positron emission tomography (FDG-PET and FMISO-PET) were used to assess metabolic tumour response and hypoxia. In all, 16 patients were accrued. Dose-limiting toxicities occurred in one patient at level 2 (grade 3 chest infection), and two patients at level 3 (grade 3 diarrhoea). Dose level 2 was declared the recommended dose level. FDG-PET imaging showed metabolic responses in 11 of the 12 primary tumours assessed. Four of six tumours had detectable hypoxia on FMISO-PET scans. The addition of oxaliplatin to infusional 5-FU chemoradiotherapy was feasible and generally well tolerated. For future trials, oxaliplatin 85 mg m^−2^ and 5-FU 200 mg m^−2^ day^−1^ continuous infusion 96 h week^−1^ is the recommended dose when combined with 50.4 Gy of pelvic radiotherapy.

In the treatment of rectal cancer, concurrent chemoradiation with 5-fluorouracil (5-FU) improves both local control and overall survival when compared with radiotherapy alone. Combined modality treatment has been accepted as an integral part of the pre- and postoperative treatment of rectal cancer (Pahlman and Glimelius, 1990; Tveit *et al*, 1997). While advances in the planning and application of radiotherapy have been made, as yet there have been only small gains in chemotherapy. Protracted venous infusion of 5-FU has been proven superior to bolus 5-FU when combined with radiation (Krook *et al*, 1991; O'Connell *et al*, 1994). Oxaliplatin is a novel platinum agent that has considerable activity in colorectal cancer. With 5-FU and folinic acid, the combination is synergistic producing response rates over 50% (Goldberg *et al*, 2004), leading to significant disease progression-free advantage in the metastatic setting. Like other platinum derivatives, oxaliplatin may also enhance tumour radioresponsiveness (Blackstock *et al*, 2000). Based on the synergism with 5-FU and its potential for enhanced radiosensitisation, oxaliplatin could increase the efficacy of infusional 5-FU when combined with radiation in the treatment of locally advanced or inoperable rectal cancer. This combination could theoretically provide superior local and systemic control of disease, and thereby improve palliation and quality of life.

We conducted a two-centre, open-label, nonrandomised dose escalation study investigating the feasibility of oxaliplatin combined with concomitant 5-FU and radiotherapy in inoperable or metastatic rectal cancer patients. The objective of this phase I trial was to define the recommended doses of oxaliplatin when used in this setting. Secondary exploratory objectives were assessment of response rates at primary and metastatic sites, including correlation of metabolic response by fluorine-18 fluorodeoxyglucose positron emission tomography (FDG-PET) with conventional imaging (computed tomography (CT)) response. The presence of tumour hypoxia is recognised as an adverse prognostic factor in other solid tumours treated with radiation (Nordsmark and Overgaard, 1996). We also sought to determine by fluorine-18 fluoromisonidazole positron emission tomography (FMISO-PET) if primary rectal tumours demonstrate imageable hypoxia.

## MATERIALS AND METHODS

The trial was approved by the human research ethics committees of the two participating centres, Peter MacCallum Cancer Centre and Austin and Repatriation Hospital.

### Eligibility criteria

Patients entering the study had histologically proven rectal adenocarcinoma, either T3, T4, M0 deemed unresectable or any T M1 requiring local treatment at diagnosis. No previous chemotherapy or radiotherapy was allowed. The patient's tumour was to be within 15 cm of anal verge. Patients were to be 18 years or older, with an Eastern Cooperative Group (ECOG) score of 2 or less. Haematologic, renal and liver function had to be adequate: absolute neutrophil count ⩾1500 mm^−3^, haemoglobin ⩾100 g L^−1^, platelets ⩾150 000 mm^3^, serum creatinine ⩽1.25 × upper limit of normal, bilirubin ⩽2.0 × upper limit of normal and liver transaminases less than twice the upper limit of normal. Written informed consent was required. Patients were excluded from the trial if they had a history of myocardial infarction within the previous 6 months or unstable cardiac disease, history of other malignancy (except nonmelanocytic skin cancer) peripheral neuropathy, central nervous system metastases, pregnant or lactating, psychiatric disorders or being treated with other investigational agents.

### Pretreatment evaluation

Pretreatment evaluation consisted of biopsy, digital rectal examination, rigid sigmoidoscopy, ECOG performance status, complete medical history and physical examination 14 days prior to first dose of the study drug. Complete laboratory results, abdominal and pelvic CT and chest X-ray were also required. Some participants were also assessed by FDG-PET and FMISO-PET scans.

### Radiotherapy

Pelvic radiotherapy was given with a megavoltage machine (6–18 MV), using a three- or four-field technique. The patient was placed in prone position and a bellyboard was utilised to minimise the amount of small bowel in the treatment field. The total radiation dose was 50.4 Gray (Gy) in 1.8 Gy per fraction per day, 5 days per week. The first 45 Gy was given to the pelvic field. The final 5.4 Gy was given to a boost field encompassing any gross disease with a 2 cm margin. The pelvic field was treated to a planning target volume with upper border at the L5/S1 junction, inferior border 3 cm below the primary tumour or at the inferior aspect of the obturator foramina, whichever was the most inferior, lateral border at 1.5 cm lateral to the widest bony margin of the true pelvic side wall and posterior border at a minimum of 1 cm behind the anterior bony sacral margin. Treatment planning was performed with computerised dosimetry. All fields were treated daily.

### Chemotherapy

Oxaliplatin (*Exolatin*™, Sanofi∼Synthelabo) was administered as a 2-h infusion on day 1 of weeks 1, 3 and 5 of radiotherapy. Dose level 1 was 70 mg m^−2^, and dose levels 2 and 3 was 85 mg m^−2^. Infusional 5-FU was given as a protracted venous continuous infusion via a peripherally inserted central catheter or implantable device line by an ambulatory pump for 96 h (5 days week^−1^) at the first two dose levels, or for 168 h (7 days week^−1^) at the third dose level. The dose was 200 mg m^−2^ day^−1^. Patients could only receive the oxaliplatin if they had ⩾1500 mm^−3^ neutrophils and ⩾150 000 mm^−3^ platelets.

### Patient monitoring during study treatment

Patients were reviewed weekly during the course of chemoradiation. Full haematological and biochemical profile were assessed weekly during treatment. Toxicity was recorded by worse grades experienced regardless of relationship to study drugs. Adverse events were graded according to National Cancer Institute Common Toxicity Criteria (NCIC version 2.0, 1999). Colony-stimulating factors or other anticancer medication were not permitted during the study.

### Post-treatment follow-up

Post-treatment evaluations were performed at 4 weeks, 3 months and 6 months after chemoradiation to document progress and toxicity. Follow-up included clinical history, examination, haematology and blood biochemistry assessments. Response was assessed 4 weeks after the completion of chemoradiotherapy according to World Health Organization (WHO) criteria by conventional abdominal and pelvic CT, and also for some by FDG-PET. Patients with potentially resectable disease following treatment could proceed to surgery 4 to 6 weeks after completion of chemoradiotherapy.

### Study design, end points and definitions

This was a phase I dose escalation study, with toxicity being the major end point. The aim of this study was to assess the tolerability and feasibility of combining oxaliplatin with concomitant 5-FU chemoradiation. The primary objective was to find a suitable oxaliplatin dose level that could be recommended for subsequent phase II trials.

Four dose level combinations were planned. Oxaliplatin was planned at 70, 85 or 100 mg m^−2^. The highest administered dose was the level at which two or more of the six patients experienced dose-limiting toxicities (DLTs). The recommended dose was the level below.

DLTs were defined as toxicity experienced during, and within 2 weeks following chemoradiotherapy. These were defined as: grade 4 neutropenia (ANC<0.5 × 10^9^ l^−1^) ⩾5 days, grade 4 thrombocytopenia (platelet count <10 × 10^9^ l^−1^), grade 3 thrombocytopenia (platelet count 10–49 × 10^9^ l^−1^) with bleeding, febrile neutropenia (38.5°C or higher for more than 24 h and neutrophils <500 mm^−3^), grade 3 or 4 nonhaematological toxicity outside the irradiated volume (excluding alopecia, grade 3 emesis and anaemia), grade 3 lower gastrointestinal or genitourinary toxicity or any grade 4 toxicity within the irradiation volume, or interruption of radiotherapy for more than 1 week.

The rules of progression to the next dose level were: at least three patients entered at each dose level. If no DLTs occurred, escalation to the next dose level occurred. If there was one DLT in the three patients, three additional patients were entered at this dose level. If ⩾2 of the six (two out three) patients had a DLT, no further dose escalation took place.

### FDG-PET and FMISO-PET imaging

Secondary objectives were to assess response at primary and metastatic sites by CT and FDG-PET imaging. FDG-PET parameters included maximum standard uptake value (SUV) and visual response score (qualitative metabolic responses), which were graded as a complete metabolic response (CMR) or partial metabolic response (PMR) by experienced nuclear medicine physicians. A CMR was assigned for cases where activity within the primary tumour site had decreased to be equal to or less than adjacent soft tissue in the radiation treatment volume. Areas of increased uptake in bowel segments proximal and distal to the primary tumour site that demonstrated higher uptake than that corresponding to the primary lesion were interpreted as radiation proctitis. FDG-PET scans were performed after a fast of at least 6 h, with imaging at least 60 min after radiotracer administration. Positron emission tomography scans for tumour hypoxia (^18^F misonidazole) were performed at baseline and postcompletion of study protocol in six patients. FMISO-PET scans were obtained 2 h after radiotracer administration. All PET imaging was performed on a dedicated PET scanner (PENN-PET 300H; UGM Medical System Inc., Philadelphia, PA, USA), with the data processed using measured attenuation correction and iterative reconstruction. Paired FDG- and FMISO-PET scans were coregistered.

FMISO-PET scans were interpreted qualitatively after coregistration with the baseline FDG-PET scan. Tumours were classified as having evidence of hypoxia if the site of known tumour on FDG imaging had higher FMISO uptake than both cardiac blood pool and normal bowel activity. It should be noted that there is colonic excretion of this radiotracer that can render qualitative interpretation and assignment of regions of interest for SUV analysis difficult.

### Statistical methods

The response rates were calculated as percentages of all evaluable patients who commenced treatment. In all, 95% confidence intervals (CI) for response rates were estimated using the exact probabilities of the binomial distribution. The vital status of each patient was taken to be the vital status on the close-out date. Overall survival was measured from the date of commencing protocol treatment to the date of death from any cause. Progression-free survival was measured from the date of commencing protocol treatment to the date of first progression (local, regional or distant) or death without previous progression. Survival times were censored at the close-out date for patients who were still alive.

Kaplan–Meier product-limit method was used to estimate overall and progression-free survival. In all, 95% CI for the percentage surviving at a particular time were calculated using the logit transformation. S-plus statistical software was used for the analysis.

## RESULTS

In total, 12 males and four females with a median age of 65 years were entered on this trial. In all, 15 patients (94%) had metastatic disease and two patients (13%) had T4 rectal tumours. Patient characteristics are listed in Table 1. The median follow-up time period for all patients from the date of commencement of protocol treatment was 17 months (range, 4–35 months).

### Acute toxicity

Table 2 lists all the toxicities for the 16 patients treated. There were three patients on dose level 1, seven patients on dose level 2 and six patients on dose level 3.

At dose level 1, one patient went into urinary retention, which was thought probably related to treatment. This patient required catheter insertion.

At dose level 2, one patient was admitted with a grade 3 chest infection, not associated with neutropenia. This patient required intravenous antibiotics in hospital and recovered quickly over a 48-h period. This patient subsequently developed haematemesis and melena associated with over anticoagulation with warfarin. Despite normalisation of coagulation parameters, he had a sudden cardiorespiratory arrest and died. No autopsy was performed. Although it was unclear whether the toxicity was directly related to treatment, a decision was made to consider this a DLT and expand the cohort at this level. An additional patient was admitted with a small bowel obstruction at dose level 2 after receiving 25 Gy. At laparotomy, the obstruction was found to be due to extensive peritoneal disease outside the radiation field. This was thought to be unrelated to treatment, and was hence not deemed to be a DLT. He was taken off study and did not receive any further radiation. As this patient received only 25 Gy, it was decided to treat a seventh patient on this dose level to ensure that there were six patients who had completed treatment and were evaluable for toxicity. No further DLTs were seen.

At dose level 3, two patients experienced DLTs. Two patients had grade 3 diarrhoea and grade 3 dehydration. These resolved following brief interruption of treatment and intravenous fluids. Grade 3 nausea (one patient), vomiting (one patient), fatigue (one patient), dysuria (two patients) and neurological parasthesia (one case) were also reported. These toxicities quickly resolved. No further escalation took place after dose level 3. Dose level 2, oxaliplatin 85 mg m^−2^ and a 96-h continuous infusion 5-FU at 200 mg m^−2^ per 24 h period, was deemed to be the recommended dose level.

### Treatment delivery

Out of 16 patients, 14 received a total radiotherapy dose of 50.4 Gy with concurrent chemotherapy, one patient received 45 Gy and chemotherapy and one patient received 25 Gy and chemotherapy. One patient required a 2-day break from radiotherapy due to perirectal pain. This was subsequently attributed to an anal fissure. The infusional 5-FU was also ceased temporarily. The oxaliplatin and infusional 5-FU doses were reduced in the two patients experiencing grade 3 diarrhoea.

### Efficacy

In all, 14 patients were eligible for assessment of response. Two patients died within 2 weeks of completing the protocol, and hence were not assessable. By CT criteria, in the radiation field, one patient (7%) achieved a complete response (CR), 12 patients (86%) had stable disease and one patient (7%) was not evaluable. On clinical examination, one other patient had a CR but stable disease by CT criteria. Outside the radiation field, there was one CR, one partial response (PR) (response rate 15%; 95% CI, 2%, 45%), two progressed and the rest had stable metastatic disease by CT criteria.

We performed FDG-PET studies in 12 patients to explore the utility of FDG-PET imaging in assessing response (Table 3). All patients had FDG uptake in their primary tumours before chemoradiotherapy. On qualitative FDG-PET visual responses, 11 of 12 patients had a response. There were six PMR, five CMR and one was reported as no change. The mean baseline SUVmax before treatment (SUV1) was 9.73 (range, 5.2–14.1) and the post-treatment SUVmax (SUV2) averaged 3.6 (range, 1.4–7.35). The mean percentage reduction in SUV values ([SUV1−SUV2/SUV1] × 100%) in the primary tumours was 61.1% (range, 32.8–100%). Of the five patients who proceeded to surgery, two had documented FDG-PET CMR and three PMR. However, all five had residual microscopic disease pathologically.

With regard to the metastatic sites of disease, generally the largest lesion was chosen for FDG-PET assessment (*n*=11). All but one known metastatic lesion was FDG avid, with a mean SUV1 of 7.0 (range, 4.7–10.2) pretreatment and a mean SUV2 post-treatment of 5.5 (range, 2.6–8.1). The mean percentage reduction for the metastatic lesions was 24.5% (range, 6.3–39%). By visual response score, there were no CMR, four PMR and six reported as unchanged. At the time of last follow-up, five of six patients without a qualitative change in FDG uptake at metastatic sites had died *vs* one of four who achieved a PMR.

Table 3 correlates FDG-PET and CT responses. While the majority of rectal lesions were reported as unchanged on CT criteria, all but one had definite metabolic responses on PET. In one patient, there was evidence of progression in the primary tumour on CT, but a PMR on FDG-PET. However, when the CT was repeated 4 weeks later, it confirmed a responding lesion. Both patients who had CRs on clinical examination or CT criteria also achieved a CMR on FDG-PET.

### Tumour hypoxia

Six patients were assessed for tumour hypoxia by baseline FMISO-PET imaging using qualitative scoring. Four of six primary tumours had detectable hypoxia at baseline scanning. Of the metastatic lesions, five of six were hypoxic. One patient had a nonhypoxic primary and hypoxic bone metastases on FMISO-PET imaging. Owing to adjacent activity in bowel, it was difficult to define a discrete region of interest for SUV calculation with the area of presumed tumoral uptake based on coregistration of the FDG and FMISO studies often overlapping with normal colonic activity. Accordingly, qualitative or semiquantitative analysis of FMISO uptake in this region is likely to be significantly compromised without reference to either anatomical (PET/CT) or metabolic (FDG-PET) characterisation of tumoral limits and its relationship to the bowel lumen.

### Surgery

Five patients underwent surgery postchemoradiotherapy treatment. Four patients could be completely resected, one patient had involved margins. There were no pathological CRs seen. Of the five patients, four had abdominal–perineal resections, and one had a Hartmann's procedure. One patient had a pelvic abscess postoperatively requiring examination under anaesthesia and surgical drainage. There were no other significant complications postresection.

### Survival

The median overall survival was 12 months (95% CI, 8 to >18 months) with 88%(95% CI, 61–97%) surviving at 6 months and 45% (95% CI, 19–75%) surviving at 1 year. The median progression-free survival time was 6 months (95% CI, 3–8 months) with 56% (95% CI, 32–72%) and 8% (CI, 1–41%) surviving without progression at 6 and 12 months, respectively.

## DISCUSSION

The addition of oxaliplatin to standard chemoradiotherapy with 5-FU was feasible, well tolerated and active in treating inoperable and metastatic rectal cancer in this phase I trial. The acute toxicities were acceptable and there were no cases of febrile neutropenia. Most patients experienced tolerable neurotoxicity, mainly of grade 1 severity, with one grade 3 case observed at the third dose level. Dose level 2 of oxaliplatin at 85 mg m^−2^ day 1, 22 and 43 during radiotherapy combined with a 96-h continuous infusion of 200 mg m^−2^ 5-FU day^−1^ was considered the recommended dose.

Combined postoperative chemoradiotherapy with 5-FU has been shown to decrease local recurrence rates and improve survival in patients with Dukes' B2 and C rectal cancer (Moertel, 1994). Preoperative chemoradiation may result in less acute and long-term toxicity and lower recurrence rates than postoperative treatment (Camma *et al*, 2000; Sauer *et al*, 2004). Preoperative radiotherapy may also permit subsequent surgery in tumours that were initially unresectable or allow sphincter sparing surgery (Swedish Rectal Cancer Trial, 1997). The addition of oxaliplatin to radiation and 5-FU could improve the effectiveness of preoperative chemoradiation, that is, increase the likelihood of downstaging inoperable tumours, and sphincter sparing surgery could also be better facilitated. For T4 tumours, results with conventional chemoradiation with 5-FU are often unsatisfactory, and this group has a high risk of developing distant disease (Janjan *et al*, 1999). In patients with metastatic disease at diagnosis and local symptoms, a regimen that could provide effective local and systemic control, without significantly increasing the toxicity profile would be good palliation in a group whose prognosis is poor.

With regard to efficacy, although not the primary end point, we have obtained some preliminary data about the response in both the metastatic and primary lesions by FDG-PET and CT imaging (Table 3). Response of rectal cancers to treatment can be difficult to assess by conventional restaging. In the 12 patients who were assessed by FDG-PET, all had uptake in their primary tumour. This is consistent with the literature suggesting that most colorectal primaries are FDG avid (Blahd *et al*, 1996). FDG uptake was also seen in all but one known metastatic site.

Evaluation of therapeutic response on FDG-PET is not yet well validated. The difficulties posed by semiquantitative measures of FDG uptake such as the SUV is that activity in adjacent soft tissue and bowel may increase following radiotherapy, presumably reflecting an inflammatory response. Accordingly, we also used a visual qualitative response score that incorporated an evaluation not only of the intensity but also the pattern of uptake. Using a threshold of a 25% reduction in SUV proposed by the EORTC (Young *et al*, 1999), all 12 primary lesions would have been classified as responding. On qualitative response scoring, there were 11 out of 12 FDG-PET responses in the primary tumours postcompletion of chemoradiation, compared to two out of 14 by conventional restaging. The nonresponder on visual criteria had an SUV reduction of 32.8%.

There is little in the reported literature about the prognostic value of FDG-PET in rectal cancer. In one study, the post-treatment SUV2 value was found to be more important prognostically, rather than the SUV percentage reduction. Patients with a primary tumour SUV2 greater than 3.2 after chemoradiation were more likely to relapse (Oku *et al*, 2002). As with our previous experience with the use of FDG-PET for therapeutic monitoring in lung cancer (Kalff *et al*, 2001), the current study demonstrated that a local CMR on PET was more common in the primary tumour than a CR on conventional imaging. Although we have shown that a CMR is prognostically significant in lung cancer patients (Mac Manus *et al*, 2003), the lack of CMR in the metastatic sites, which were present in the majority of patients, limits assessment of the prognostic significance of a local CMR in our cohort. Although both patients who achieved a local CMR and subsequently had surgical resection still had residual miscroscopic disease, this is not unexpected given the limitations of all imaging modalities to detect small volume disease. Furthermore, it does not necessarily imply that a CMR will not confer a prognostic advantage compared to patients with only a partial or no metabolic response. Further studies assessing the prognostic significance of a local CMR in patients without disease outside the radiation treatment volume are required to validate this technique as an objective evaluation of local response. Nevertheless, our preliminary data suggest that FDG-PET may provide useful information regarding both local and systemic response in combined modality therapies.

Our FMISO-PET imaging results demonstrate that hypoxia can be detected in some rectal cancers. There are few published studies in this area (Wendling *et al*, 1984; Molls *et al*, 1994), none using functional imaging, despite hypoxia being well established in other malignancies as a significant obstacle to successful treatment with radiation (Nordsmark *et al*, 1996). Technical limitations related to high background activity in the normal colon need to be addressed if semiquantitative evaluation of tumoral uptake were required. Even for qualitative evaluation, clear delineation of the tumour location is required. For our study, coregistration with an FDG-PET study was used for this purpose, but with the advent of combined PET/CT scanners, documentation of specific tumoral uptake may be enhanced.

While the responses of the metastatic lesions seemed low by conventional criteria, this was likely influenced by the cessation of the treatment at 5 weeks, early restaging and the low cumulative oxaliplatin dose received. In the original study by de Gramont (de Gramont *et al*, 2000), which compared leucovorin and 5-FU with or without oxaliplatin in the first-line setting for advanced disease, the median time to response was seen at 9 weeks in the oxaliplatin combination arm, and the oxaliplatin dose used was higher. Other chemoradiation studies have varied in their cumulative oxaliplatin dose. Freyer *et al* (2001) used oxaliplatin only on days 1 and 29 of treatment using dose escalations of 80, 100 and 130 mg m^−2^ with the MTD not being reached. Rodel *et al* (2003) used a cumulative dose of 240 mg m^−2^, with oxaliplatin administered days 1, 8, 22 and 29. Grade 3 diarrhoea was the DLT; the recommended dose was 60 mg m^−2^. Aschele *et al* (2002) administered oxaliplatin 60 mg m^−2^ weekly with radiotherapy. Grade 3 diarrhoea was the DLT, and 64% experienced grade 1 or 2 neurotoxicity. Another study found the MTD of oxaliplatin to be 150 mg m^−2^ given days 2 and 30 with 5-FU and radiotherapy, with DLTs of grade 3 diarrhoea and neurological toxicity (Sebag-Monefiore *et al*, 2002). Maintenance chemotherapy postcompletion of chemoradiation should be explored in future studies in those who show sensitivity to treatment as the higher cumulative doses received may increase response rates at distant sites.

Our study is comparable to the results of other phase I and II trials (Freyer *et al*, 2001; Carraro *et al*, 2002; Sebag-Monefiore *et al*, 2002) assessing this combination, predominantly in the locally advanced setting. There was no apparent increase in complications seen in the five patients who underwent surgical resection compared to other studies (Francois *et al*, 1999). No significant late toxicity has been observed though follow-up is short.

In conclusion, oxaliplatin combined with 5-FU infusional chemoradiotherapy is feasible and well tolerated. The recommended doses are oxaliplatin at 85 mg m^−2^ days 1, 22 and 43 combined with a 96-h continuous infusion 5-FU each week and concomitant radiotherapy. In addition to a potential role in the preoperative setting for inoperable or locally advanced rectal cancer, patients with metastatic disease and local symptoms may benefit from this approach, and schedules including further cycles of chemotherapy should be explored.

## Figures and Tables

**Table 1:** Patient characteristics

	**Patients**	
**Characteristics**	**No.**	**%**
*Age*		
Median	65 years	
Range	32–81 years	

*Sex*		
Male	12	75
Female	4	25

*ECOG performance status*		
0	1	6
1	10	63
2	5	31

*Histology*		
Well differentiated	1	6
Moderately differentiated	8	50
Poorly differentiated	2	13
Mixed differentiation	3	19
Not specified	2	13

*Stage*		
T3	13	81
T4	2	13
Tx	1	6
N0	2	13
N1	7	44
N2	6	38
M0	1	6
M1	15	94

*Metastatic disease*		
Nil	1	6
Bone	1	6
Liver	11	69
Lung	5	19

ECOG=Eastern Cooperative Group.

**Table 2:** Toxicities in the Study Population (*n*=16)

	**Dose level 1 (*n*=3)**				**Dose level 2 (*n*=7)**				**Dose level 3 (*n*=6)**			
**Toxicity**	**Oxaliplatin 70 mg m^−2^; 96 h CI 5-FU**				**Oxaliplatin 85 mg m^−2^; 96 h CI 5-FU**				**Oxaliplatin 85 mg m^−2^; 168 h CI 5-FU**			
**Grade**	**1**	**2**	**3**	**4**	**1**	**2**	**3**	**4**	**1**	**2**	**3**	**4**
*Haematologic*												
Leukopenia	1	0	0	0	1	3	0	0	2	1	0	0
Neutropenia	1	0	0	0	1	2	0	1	0	1	0	0
Thrombocytopenia	0	0	0	0	1	0	1	0	1	0	0	0
Anaemia	2	1	0	0	3	2	1	0	3	1	0	0
Infection (without neutropenia)	0	0	0	0	0	0	1	0	0	0	0	0

*Neurosensory*												
Parasthesia/dysthesia	2	0	0	0	6	1	0	0	4	0	1	0

*Gastrointestinal*												
Nausea	2	1	0	0	5	0	1	0	2	1	0	0
Vomiting	1	0	0	0	2	0	1	0	0	0	0	0
Diarrhoea	2	0	0	0	1	5	0	0	3	1	2	0
Constipation	0	0	0	0	0	2	0	0	3	2	0	0
Stomatitis	0	0	0	0	1	1	0	0	2	0	0	0
Proctitis	0	1	0	0	3	0	0	0	1	1	0	0

*Genitourinary*												
Bladder spasms	1	1	1	0	1	0	0	0	1	0	0	0
Dysuria	0	2	0	0	4	1	0	0	0	3	2	0
Urinary frequency/urgency	1	2	0	0	4	2	0	0	0	1	0	0

*Other*												
Fatigue, malaise	1	1	0	0	2	2	0	0	3	1	1	0
Radiation dermatitis	1	0	0	0	3	1	0	0	1	5	0	0
Dehydration	0	0	0	0	1	1	0	0	0	0	1	0

5-FU=5-fluorouracil; CI=confidence interval.

**Table 3:** FDG-PET and CT imaging responses (*n*=12)

**Patient No.**	**Site of disease assessed**	**Pretreatment SUV1**	**4 weeks post-treatment SUV2**	**% reduction (1-SUV2/SUV1)**	**Qualitative visual response**	**CT response**	**Status**
1	Primary	11.39	6.9	39.4	PMR	SD	DoD
	Liver	5.67	6.55		UN	PD	4mo

2	Primary	6.43	2.99	53.5	PMR	SD	DoD
	Liver	7.92	5.33	32.7	PMR	SD	9mo

3	Primary	12	3.1	74.2	PMR	SD	DoD
	Liver	10.2	8.1	20.5	UN	SD	24mo

4	Primary	9.6	3.6	62.5	PMR	SD	DoD
	Para-aortic node	7.9	4.8	39.2	UN	PR	12mo

5	Primary	5.2	1.4	73.1	CMR	CR	AWD
	Liver	4.7	4.4	6.3	PMR	SD	

6	Primary	14.1	7.35	47.9	PMR	SD	NED

7	Primary	10	4.5	55	CMR	N/E	AWD
	Liver	5.2	4.3	17.3	PMR	CR	

8	Primary	13.6	5.41	60.2	CMR	SD	AWD
	Liver	NU	NU			SD	

9	Primary	7.33	NU	100	CMR	SD	AWD
	Bone	4.76	2.61	45.1	PMR	SD	

10	Primary	6.8	1.5	77.9	CMR	N/E^a^	DoD
	Liver	8.7	6.2	28.7	UN	SD	11mo

11	Primary	13.8	5.9	57.2	PMR	PD	AWD
	Liver	8.4	7.8	7	UN	SD	

12	Primary	7.3	4.9	32.8	UN	SD	DoD
	Liver	6.7	5.1	23.8	UN	SD	14 mo
	Lung	2.6	2.4	7.7	UN	SD	

FDG-PET=Fluorine-18 fluoro-deoxyglucose positron emission tomography; CT=computed tomography; SUV: standardised uptake value; ND: not done; NU: no uptake; CMR: complete metabolic response; PMR: partial/incomplete metabolic response; PD: progression; N/E: not evaluable. UN: unchanged metabolic response; AWD: alive with disease; DOD: dead of disease; NED: no evidence of disease; mo: months.

a CR observed on clinical examination.
